# Contraceptive use and unmet need for family planning among HIV positive women on antiretroviral therapy in Kumasi, Ghana

**DOI:** 10.1186/1472-6874-14-126

**Published:** 2014-10-11

**Authors:** Dennis Odai Laryea, Yaw Ampem Amoako, Kathryn Spangenberg, Ebenezer Frimpong, Judith Kyei-Ansong

**Affiliations:** Public Health Unit, Komfo Anokye Teaching Hospital, PO Box 1934, Kumasi, Ghana; Directorate of Medicine, Komfo Anokye Teaching Hospital, Kumasi, Ghana; Polyclinic Directorate, Komfo Anokye Teaching Hospital, PO Box 1934, Kumasi, Ghana; National AIDS/STI Control Programme, Komfo Anokye Teaching Hospital, Kumasi, Ghana

**Keywords:** Contraception, Unmet need, Ghana, HIV positive women

## Abstract

**Background:**

A key strategy for minimizing HIV infection rates especially via reduction of Mother– to-Child transmission is by reducing the unmet need for family planning. In Ghana, the integration of family planning services into Antiretroviral Therapy services for persons living with HIV/AIDS has largely been ignored. We set out to measure the prevalence of modern methods of contraception, the unmet need for family planning and to identify factors associated with the use of modern methods of contraception among HIV positive women on anti retroviral therapy.

**Methods:**

This was a descriptive cross sectional study of HIV positive women in their reproductive ages accessing care at an adult Antiretroviral Therapy Clinic in Kumasi, Ghana. Data was collected using a structured questionnaire. Data analysis was conducted using Epi Info version 7.1.2.0.

**Results:**

A total of 230 women were included in the study. Fifty six percent were in the 30–39 year age group. The mean age (SD) was 36.3 (5.4) years. While 53.5% of respondents desired to have children, partner desire for children was reported by 54.6% of respondents with partners. About 74% had received information on contraception from their provider. 42.6% of participants and/or their partners were using a contraception method at the time of study; the male condom (79.6%) being the most commonly used method. The estimated unmet need for contraception was 27.8%. Contraceptive use was strongly associated with partner knowledge of HIV status (AOR = 3.64; 95% CI 1.36–9.72; p = 0.01) and use of a contraceptive method prior to diagnosis of HIV (AOR = 6.1; 2.65–14.23; p < 0.001).

**Conclusion:**

Contraceptive Prevalence is high among HIV positive women in Kumasi compared with the general Ghanaian population. Despite this, there still is a high unmet need for family planning in this population. We recommend continuous education on contraceptives use to HIV patients accessing HAART services to further increase contraceptive uptake.

## Background

The advent of Highly Active Antiretroviral Therapy (HAART) has resulted in significant improvements in the health of persons living with the Human Immunodeficiency Virus (HIV) [1]. Globally, an estimated 34 million persons are living with HIV [2] with about 9.7 million people on HAART at the end of 2012 [3]. In Ghana, an estimated 59,000 out of the 270,000 persons living with the virus are on antiretroviral therapy (ART) [4, 5]. Provision of ART services in Ghana started in 2003 through the National AIDS/STI Control Programme (NACP) [5, 6].

Contraception and family planning are key to improving the health of a population because of the associated benefits [7]. Women on HAART are at increased risk of conception because of improved immunity and physical health, which may lead to more frequent sexual intercourse. Although Ghana’s HIV prevalence, fertility and population growth rates are relatively low compared to other sub-Saharan African nations, the rather low contraceptive prevalence rate of 25% despite an almost universal knowledge on at least one method of contraception [7, 8] presents a challenge to improving the reproductive health outcomes of persons living with HIV (PLHIV). Contraceptive use among HIV positive persons plays a crucial role in meeting their reproductive health needs- especially among discordant couples. Unmet need for family planning is one of the indicators for monitoring progress towards achieving the United Nations’ Millennium Developments Goal (UNMDG) [9]. Ghana has a high unmet need for family planning (currently estimated at 35%) among married women [7] and a similar picture may be present among HIV positive women in Ghana.

Reducing the unmet need for family planning to zero is a key component of the global fight against new HIV infections [10]. Women with unmet need are those who are fecund and sexually active but are not using any method of contraception, and report not wanting any more children or wanting to delay the next child [11]. One potential benefit of reducing the unmet need for family planning among HIV positive women is a reduction of the risk and incidence of mother-to-child transmission (MTCT) of HIV infection. Modern methods of contraception such as sterilisation, intrauterine devices, male and female condoms are effective means of preventing unwanted pregnancies. Correct and consistent condom use confers dual protection (preventing unwanted pregnancy and sexually transmitted infection) when used alone or with another non-barrier method of contraception. The practice of dual contraception may not be common [12–14]. To date no contraceptive method is absolutely contraindicated based on one’s HIV status; therefore persons with HIV are eligible for one contraceptive method or another [15]. Despite this, high rates of unintended pregnancies have been found among some HIV positive women on ART [16]. Large variations in the proportion of unintended pregnancies within a country has also been reported [17, 18]. The use of condoms among patients on HAART has been associated with fewer unintended pregnancies and lower risk of sexual transmission of HIV [19].

Fertility desire among HIV positive women are influenced by several factors. These include age, marital status and a history of child mortality [18, 20]. The age range 23–34 years, in particular, has been associated with high fertility desire. Educational level has been strongly associated with the desire to be pregnant [17]. HAART has been found to have no influence on the fertility desires of HIV positive women [18, 21, 22]. In contrast, HAART has been associated with contraceptive use among HIV positive persons, with a lower usage among HIV positive women not on ART [19]. Contraceptive use is reported to be low even among women with no desire to be pregnant [17, 23], suggesting a high unmet need for contraception. The high fertility desire among some sections of HIV positive population calls for effective strategies to increase contraceptive uptake.

We set out to estimate the contraceptive prevalence rate and the unmet need for family planning among HIV positive women in the reproductive ages on HAART in Kumasi, and to examine factors associated with the use of modern methods of contraception in this population.

## Methods

This was a descriptive cross sectional study conducted at the Adult HIV clinic of the Komfo Anokye Teaching Hospital (KATH). KATH is the referral centre for the middle/northern belt of Ghana, located in the city of Kumasi, the capital of the Ashanti Region. Kumasi is the second largest city in Ghana with an estimated population of 2.1 million [24]. The KATH adult HIV Clinic is one of the largest in Ghana and has since its inception seen cumulatively over ten thousand individual clients. The clinic runs bi-weekly sessions. Some patients come from outside Kumasi and even other regions beyond the Ashanti region. The majority of patients seen at the clinic are however resident in Kumasi.

Inclusion criteria; females aged 15–49 years, who had attended the clinic at least once during the prior six months. Eligible clients were selected through simple random sampling of the clinic folders. The next date of clinic attendance was identified to contact and invite to participate.

A total of 1,092 eligible females were identified. Based on Ghana’s current contraceptive prevalence rate of 25.2%, a sample size of 229 was estimated using Epi Info version 7.1.2.0 at a 95% confidence interval.

The data collection instrument was a structured questionnaire administered by two trained research assistants. The structured questionnaire covered demographic information, sexual partner information, history of sexually transmitted infections (any genital ulcer or discharge during the preceding year), fertility desire of both woman and partner, and disclosure of HIV status. It also included information on contraception (current and previous use) and knowledge on contraception. The duration of ART was obtained from the clinical notes of respondents. Eligible respondents were approached on their clinic days for participation in the study. Nine selected respondents declined participation and the next set of random numbers and corresponding clients were approached for participation. Following written informed consent, respondents were interviewed between December 2012 and March 2013. Ethical approval for the study was from the KATH/Kwame Nkrumah University of Science and Technology Committee of Human Research, Publications and Ethics.

Data collected was entered into a Microsoft Access Database. Statistical analysis was undertaken using Epi Info version 7.1.2.0. Univariate analysis involved the estimation of frequencies, proportions and means. The Unmet need for contraception was estimated based on the proportion of women with no desire to become pregnant but who were currently on no method of contraception. Bivariate analysis was used to examine the association between various variables using Chi-squared tests and Analysis of Variance (ANOVA). Logistic regression was conducted using the ‘current contraceptive use’ as the dependent variable and the following as the independent variables: desire to have children, partner desire to have children, previous use of a contraceptive method, history of STI, history of STI in partner and partner knowledge of HIV status. Charts were constructed using Microsoft Excel.

## Results

A total of two hundred and thirty (230) persons participated in the study. The basic demographic information of the respondents is as shown in Table 1.

**Table 1:** **Demographic characteristics of respondents (n = 230)**

Parameter	Frequency	Percentage
Age group		
20–29 years	27	11.7
30–39 years	129	56.1
40 or more	74	32.2
Marital status		
Single	36	15.7
Cohabiting	18	7.8
Married	89	38.7
Separated	19	8.3
Divorced	30	13.0
Widowed	38	16.5
Occupation		
Trading	150	65.2
Unemployed	30	13.0
Dressmaking	13	5.7
Farming	12	5.2
Others	25	10.9
Place of residence		
Kumasi	181	78.7
Outside Kumasi	49	21.3
Educational status		
Nil	113	49.1
Primary	43	18.7
Junior high/middle school	37	16.1
Secondary	28	12.2
Tertiary	9	3.9

No respondent was less than age 20 years of age. The majority of respondents (56.1%) were in the age group 30–39 years. The mean age (SD) of respondents was 36.3 (5.4) years. The majority (38.7%) of women were married. Seventy-eight percent of respondents were resident in Kumasi. About 65% of respondents were traders.

The majority of respondents had been on ART for less than 3 years. The distribution of respondents by duration on ART use is shown in Table 2.

**Table 2:** **Duration of ART among respondents**

Duration of ART use	Frequency	Percent
≤12 months	93	40.4
13–24 months	26	11.3
25–36 months	21	9.2
37–48 months	25	10.9
49–60 months	27	11.7
>60 months	38	16.5

The majority of respondents (74.8%) had no prior history of a sexually transmitted infection (STI) in the form of a genital ulcer or discharge in the one year preceding the study (Table 3). Among those with current sexual partners (n = 143), only 4.2% of respondents indicated a partner STI during the one year preceding the study.

**Table 3:** **Risk factors for HIV transmission among HIV positive women in Kumasi**

Parameter	Y/N	Frequency	Percentage
Previous history of STI in the one year preceding study	Yes	58	25.2
	No	172	74.8
	**Total**	**230**	**100.0**
Partner STI in the one year preceding study	Yes	6	4.2
	No	137	95.8
	**Total**	**143**	**100.0**
Desire for children	Yes	123	53.5
	No	107	46.5
	**Total**	**230**	**100.0**
Partner desire for children	Yes	78	54.6
	No	65	45.4
	**Total**	**143**	**100.0**

The majority of respondents (53.5%) had a desire for children of their own with partner desire for children reported by 54.6% of respondents with a partner. There was no statistically significant difference between the two groups (*p* = 0.91). Among respondents with a partner (n = 143), the desire of both partners to have a child was found among 48.6% of respondents. Fertility desires among respondents with a partner are as shown in Table 4.

**Table 4:** **Fertility desires of respondents and partners**

Desire for a child	Frequency	Percentage
Both desire	69	48.2
Woman alone	9	6.3
Partner alone	9	6.3
Both do not desire	56	39.2
Total	143	100.0

About 74% of respondents had received information on contraception during enrolment to the clinic (Table 5). These were received during the counselling sessions for testing or adherence for medication or both.

**Figure 1:**
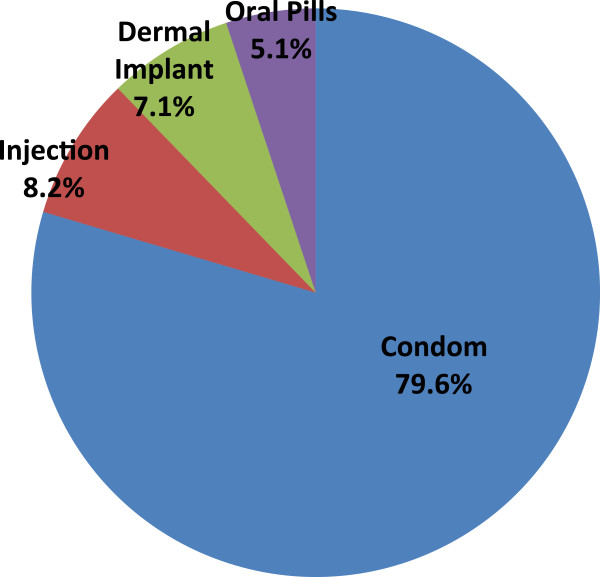
**Distribution of methods of contraception used by HIV positive women or their partners in Kumasi.**

The majority of respondents (58.3%) had previously used a method of contraception. Some 42.6% of respondents and/or their partners were currently using a method of contraception (estimated contraceptive prevalence rate). The most commonly used method of contraception was the male condom (79.6%). The other methods used were dermal implants, injectable contraceptives and oral pills (Figure 1). Respondents and their partners not using any modern method of contraception accounted for 57.4%, those using condoms accounted for 33.9% and those using other methods 8.7%.

**Table 5:** **Information on and usage of contraception among HIV positive women in Kumasi**

Parameter	Status	Frequency	Percentage
Information on contraception from health provider since diagnosis	Received	170	**73.9**
	None received/Do not remember	60	**26.1**
	**Total**	**230**	**100.0**
Previous use of any method prior to diagnosis	Yes	134	**58.3**
	No	96	**41.7**
	**Total**	**230**	**230**
Current use of a modern contraceptive method by self or partner	Yes	98	**42.6**
	No	132	**57.4**
	**Total**	**230**	**100.0**

Desire to have children, previous STI and previous STI in partner were found not to be significantly associated with contraceptive use. However, previous use of a method of contraception prior to diagnosis with HIV, partner desire for a child and partner knowledge of HIV status were found to be significantly associated with current contraceptive use.

Women whose partners desired a child were 50% less likely to use a contraceptive method (Table 6). Women who had previously used a method or whose partners were aware of their HIV status were more likely to be currently using a contraceptive.

**Table 6:** **Predictors of contraceptive use among HIV positive women in Kumasi**

Parameter	Odds ratio*	95% CI	***p***
Desire to have children	1.58	1.00–2.90	0.06
Previous STI	0.93	0.51–1.71	0.95
Partner with previous STI	0.85	0.17–4.37	0.85
Partner desire for a child	0.50	0.26–0.98	0.04
Previous contraceptive use before diagnosis	7.10	3.79–13.31	<0.001
Partner knowledge of HIV status	4.56	2.30–9.07	<0.001

*Crude OR.

Partner knowledge of HIV status (AOR = 3.64; 95% CI 1.36–9.72; p = 0.01) and previous use of a contraceptive method (AOR = 6.1; 2.65–14.23; p < 0.001) were found to be significantly associated with the use of a modern method of contraception on regression analysis. The following associations with contraceptive use were however found not to be statistically significant [partner desire for a child (AOR = 1.38, 95% CI = 0.38–5.04, p = 0.62); desire to have children (AOR = 1.21, 95% CI = 0.34–3.46,p = 0.78), previous STI (AOR = 0.86, 95% CI = 0.29–2.55, p = 0.78) and partner with previous STI AOR = 0.70, 95% CI = 0.06–8.14, p = 0.78)]. Adjusting for age and education (p = 0.33) also established no statistically significant relationship with contraceptive use.

The majority of married women (68.5%) used a method compared with 50.0% among cohabitating women and 10.3% among widows.

Condom use was found among 25.7% of all respondents and 74.7% of respondents using a method (n = 78) and 66.7% of women with no fertility desire (Table 7). Married women accounted for the majority of respondents using condoms as a method of contraception (54.2%).

**Table 7:** **Methods of contraception used among women with no fertility desire**

Contraceptive type	Frequency	%
Condom	22	66.7
Dermal implant	5	6.1
Oral contraceptive	4	15.1
Injectable contraceptive	2	12.1
Total	33	100

The educational status was associated with an increased likelihood of contraceptive use on chi square test for trend from respondents with no education to those with tertiary education (OR = 1.43). This association was however found not to be statistically significant (p = 0.23). The duration of ART was also not associated with contraceptive use (Table 8).

**Table 8:** **Duration of ART and contraceptive use**

Parameter	Odds ratio	95% C.I.	P-Value
12–23mths/<12mths	1.45	0.60–3.46	0.40
24–35mths/<12mths	0.72	0.27–1.96	0.52
36–47mths/<12mths	0.81	0.33–2.03	0.65
48–59mths/<12mths	1.81	0.76–4.29	0.17
>59mths/<12mths	1.05	0.49–2.26	0.89

Across the marital status, the significant association was found comparing widowed women with cohabiting women with widowed women less likely to use a modern method of contraception compared with cohabiting women (OR = 0.11, 95% CI = 0.02–0.64, p = 0.01).

### Unmet need for family planning

**Figure 2:**
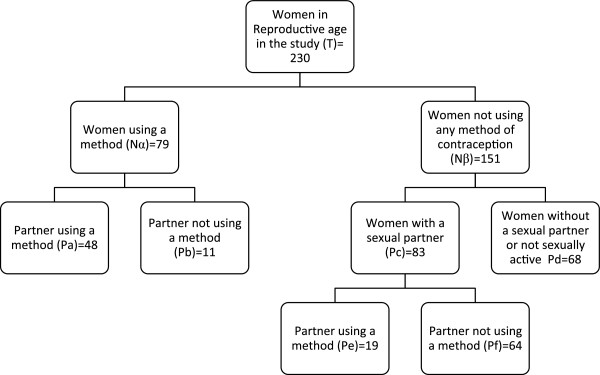
**Algorithm for contraception use and unmet need for family planning among HIV positive women in Kumasi.** ; .

The estimated unmet need for contraception in this cohort is 27.8% (Figure 2). Among women with no desire to have children (n = 107), 69.2% are not currently using a method of contraception. Condoms were the commonly used method with 66.7% (n = 22) using this method.

With regards to the educational status, increasing level of education was not associated with unmet need for contraception (p = 0.54). Compared with women who had an unmet need for family planning, the mean duration of ART was not different from those with an unmet need for family planning (p = 0.30).

## Discussion

This study recorded a contraceptive prevalence rate of 42.6% among HIV positive women attending an adult Antiretroviral treatment clinic in a referral hospital in Kumasi. The prevalence rate recorded is higher than the estimated contraceptive prevalence rate in Ghana of 25.2% among the general population [7, 8]. However, the prevalence recorded among respondents in this study is lower compared with those recorded among HIV positive persons in countries such as South Africa [19]. A higher prevalence of contraception is desirable both in the general population and among HIV positive persons in the light of the high fertility rates recorded in some parts of Africa [20, 21, 25] and the need to reduce the risk of transmission of HIV.

Some barrier contraceptives are useful for preventing unwanted pregnancies and STIs including HIV. The use of condoms has been estimated to reduce the risk of horizontal transmission of HIV significantly [26, 27]. Our finding of condoms as the most preferred method of contraception is good in light of the evidence. Condoms may be the commonly used method among the respondents in this study because of the conferment of dual protection, its ready availability over-the-counter, relative ease of use, promotion by the health authorities in Ghana and the avoidance of medication and perceived side effects of other contraceptives. Consistent condom use has been associated with an 80% reduction in HIV incidence [26]. However, the use of condoms among respondents as a choice of contraceptive is still low although it accounted for a higher proportion of the contraceptive methods used by respondents in this study. The use of other modern methods of contraception in this study was low (20.4%- dermal implants, oral pills and injectables) compared with condoms (79.6%). While a high contraceptive prevalence rate is generally desirable, the use of condoms in particular is important among HIV positive women particularly in cases of discordance. The low use of other modern methods compared with condoms among respondents in this study is therefore an important finding. The observation of a poor association between previous STI in both the respondents or in their partners and contraceptive use is a source of concern in attempts at reducing the transmission of HIV and other STIs. The presence of other STIs may facilitate HIV transmission and is of greater importance among sero-discordant couples. This makes the need for condom use even more important. Female condom use is generally low in Ghana [8] so the male condom remains the most relevant choice of barrier contraception among HIV positive persons particularly among discordant couples.

Fertility desire among some populations have been found to be equivalent to that among HIV positive women [21, 22]. Despite being HIV positive, we found more than half of respondents with the desire to have children, which is much higher than the 28.6% recorded in Uganda [18] and 31% recorded in South Africa [28]. Anti-retrovirals (ARVs) have made the outcomes of pregnancies among HIV positive women favourable, including low risk of mother-to-child transmission. Fertility desire among respondents in this study was found to be similar when married women were compared with the other categories of marital status the exception being widowed women. Widowed women had a lower desire and the difference was statistically significant. These notwithstanding, those with no fertility desires must have appropriate contraceptive services preferably, barrier methods, available to them. The high fertility desires recorded in this study may also have implications for the elimination of MTCT of HIV programmes in Ghana. Women on HAART may be at a lower risk of transmitting infection to their children but a high fertility rate means the number of HIV positive infants may also increase as a result.

Our study found that partner knowledge of HIV status and use of contraception prior to diagnosis of HIV were strongly associated with current use of contraception. This highlights the need for disclosure to be a key objective in the provision of ART services. All persons living with HIV (PLWHIV) should be encouraged as much as possible to disclose their status to their partner. This disclosure arms the couple to take informed decisions with positive outcomes. Respondents whose partners desired a child were less likely to use a method of contraception, this buttresses the importance of counselling both partners where necessary to be able to achieve a goal [29].

Although the majority of respondents had received prior information during the post-HIV test counselling and ART initiation/Adherence counselling, about a quarter of women in this study indicated that no such service was received. Despite the high level of knowledge on contraception in Ghana [8], uptake is very low and this makes it imperative to reinforce the relevance of contraceptive use at every stage of HIV care to further increase uptake of contraception and reduce the unmet need for contraception among HIV positive women.

Integrating family planning services with HIV care has been recommended [16, 19, 30, 31] as well as continuous reproductive health service provision during ART care [32]. Our study found that a high proportion of women with no desire to be pregnant were not using any modern method of contraception [17]. This high proportion of 69.2% of non-use of contraception among HIV positive women can potentially lead to unintended pregnancies and risk of transmission of HIV to infants and partners who are not HIV positive [23, 33]. Avoiding unintended pregnancies among women can reduce the risk of MTCT. Despite the lower unmet need for family planning (27.8%) in our study compared with the national estimate of 32% [7], the risk of HIV transmission makes the lower unmet need for contraception among HIV positive women desirable to reduce the risk of transmission particularly among discordant couples. Family planning services are not available at the Adult HIV clinic and this may have been a factor in our findings in this study. There may be the need to integrate family planning services in the Adult HIV clinic in Kumasi.

### Limitations

The study excluded pregnant HIV positive women as they are seen at a different clinic. This exclusion was for practical purposes and may have resulted in an under-estimation of the unmet need for family planning among respondents. Our estimation of the unmet need for contraception also assumed that all women in the study were fecund and this may have resulted in an overestimation of the unmet need for contraception. We are however convinced that the possibility of overestimation on one hand and underestimation on the other may provide some form of compensation for the possible errors in measurement.

The wide confidence interval observed for the association between previous contraceptive use and current usage may be due to the small sample size in this study. Partner use of condoms was as reported by respondents and may have been influenced by the social desirability for condom use by HIV positive persons.

## Conclusion

Contraceptive uptake is high among HIV positive women in their reproductive ages compared with the general Ghanaian population. This observation may however not be representative of the HIV positive population in Ghana and further studies on the subject is recommended. Partner knowledge of HIV status and previous contraceptive use are strong predictors of contraceptive use. A high unmet need for family planning still exists. HIV carers must provide reproductive health services at every stage of HIV care including counselling, testing, drug adherence counselling and during reviews of patients on HAART.

## Authors’ information

DOL works as a Public Health Physician in a Teaching Hospital in Ghana. He has keen interest in reproductive and child health issues particularly those in relation to the United Nations Millennium Development Goals 4, 5 and 6. He currently heads the Public Health Unit of KATH.

YAA is a Senior Specialist Physician at the Directorate of Medicine in KATH. He has extensive experience in Infectious Diseases and Tropical Medicine. He also teaches clinical medicine to medical students during their junior and senior clerkships at the Komfo Anokye Teaching Hospital in Kumasi.

KS is a Family Physician and Head of Family Medicine at the Komfo Anokye Teaching Hospital, Kumasi, Ghana. She is the Director of the Family Residency Programme at the Hospital. Her areas of interest in Family Medicine practice include women’s health issues.

EF is a Public Health officer and works with the National AIDS and STIs Control Programme. He is involved in the running of the Paediatrics and Adult HIV clinics.

JK-A is a Population and Reproductive Health Officer and currently manages HIV data for the Komfo Anokye Teaching Hospital. She has been involved in training of health staff on issues of HIV prevention and management.

## Abbreviations

AIDS: Acquired Immune Deficiency Syndrome
AOR: Adjusted Odds Ratio
ART: Antiretroviral Therapy
ARV: Antiretroviral
CI: Confidence Interval
HAART: Highly Active Antiretroviral Therapy
HIV: Human Immunodeficiency Virus
KATH: Komfo Anokye Teaching Hospital
MTCT: Mother-to-Child Transmission
NACP: National AIDS/STI Control Programme
OR: Odds Ratio
PLWHIV: Persons Living With HIV
SD: Standard Deviation
STI: Sexually Transmitted Infection
UNICEF: United Nations Children Fund
UNMDG: United Nations Millennium Development Goals
WHO: World Health Organisation.
